# CrackNet: A novel multi-scale architecture for crack segmentation

**DOI:** 10.1371/journal.pone.0346889

**Published:** 2026-04-15

**Authors:** Wubiao Zhu, Mengcai Ye, Jiawei Yin, Jingying Mo, Zhendi Ma, Ruibing Xie

**Affiliations:** 1 Zhejiang Guangsha Vocational and Technical University of Construction, Zhejiang, China; 2 ShangHai University, Shanghai City, Shang Hai, China; 3 College of Computer Science and Technology, Zhejiang Normal University, Jinhua, China; Samsun University: Samsun Universitesi, TÜRKIYE

## Abstract

Crack detection is essential for structural safety inspection but remains challenging due to noise, illumination variations, and complex backgrounds. In this paper, we propose CrackNet, a segmentation network specifically designed for concrete crack detection. CrackNet integrates three key modules: a lightweight multi-scale convolution enhancement block (LightMSCBlock) in the encoder to capture both local details and global context, a SAF attention module embedded in skip connections for scale-aware feature fusion and edge refinement, and a multi-scale feature fusion (MSFF) module in the decoder to enhance feature integration while reducing information loss. Extensive experiments on three public datasets—CFD, Crack500, and DeepCrack—demonstrate that CrackNet consistently outperforms state-of-the-art methods. Specifically, on CFD, F1 and IoU improve by 6.37% and 7.1% over SegFormer; on Crack500, F1 increases by 3.86% compared with MobileNetV3-UNet; and on DeepCrack, F1 and IoU gains reach 5.7% and 2.5%, respectively. Ablation studies further confirm the complementary effectiveness of LightMSCBlock, SAF, and MSFF. Overall, CrackNet achieves superior accuracy and robustness, showing strong potential for real-world engineering applications. The code is available at the following link: https://github.com/xzz-ya/CrackNet.git

## 1 Introduction

Surface cracks are one of the most common and significant types of damage in structural health monitoring. Timely and effective detection and assessment of these cracks are essential for ensuring the safety and durability of buildings and infrastructure [1,2]. Over time, structural materials may crack because of wear and tear, weathering, and heavy loads. If these cracks are not identified and addressed promptly, they may evolve into severe safety hazards and even lead to catastrophic failures. Traditional manual inspection methods are time-consuming, labor-intensive, and highly subjective, often resulting in low detection accuracy, which makes them inadequate for the modern engineering demand for efficient and intelligent crack identification [3]. Therefore, accurately locating crack positions and reconstructing their geometric features has become a core technical challenge for ensuring structural safety [4–7].

Recently, crack detection has mainly relied on traditional image processing and handcrafted feature extraction. These approaches typically use grayscale, texture, edge, or frequency information combined with rule-based algorithms to isolate crack regions. Although efficient and easy to implement, they are sensitive to image quality, background complexity, and variations in crack appearance, limiting robustness and generalization in real-world scenarios. The Canny algorithm is widely adopted in edge detection methods for its strong noise suppression and accurate boundary localization [8]. Subsequent improvements in Gaussian filtering and thresholding have enhanced its sensitivity and robustness [9]. Sobel and Prewitt operators have also been used to localize cracks’ edges. Morphological operations are often applied to eliminate minor artifacts and connect fragmented edges. Zhang et al. [10] combined morphological reconstruction with a distance-based shape descriptor to extract dark crack regions in complex backgrounds. Thresholding techniques also play a central role. Otsu’s method is effective when there is a strong grayscale contrast between cracks and the background [11]. Later studies incorporated grayscale discrimination and regional histogram analysis to enhance segmentation in low-contrast cases [12]. Local adaptive thresholding and unsupervised methods such as maximum entropy have also been explored. Texture-based methods use statistical descriptors to represent crack patterns. The Gray-Level Co-occurrence Matrix (GLCM), for example, captures contrast and entropy and was used by Arya et al. [13] to develop an automatic classification method. Other descriptors, including Tamura features and the Histogram of Oriented Gradients (HOG), help capture directionality and local variations [14]. Fourier and wavelet transforms enable the separation of periodic and multiscale features in the frequency domain. Ranjbar Set al. [15] proposed a hybrid method combining wavelet features with deep neural networks for improved crack detection. Due to their directional sensitivity, Gabor filters have also been effective at enhancing noisy textures [16]. Zheng R et al. [17] proposed a hybrid technique that merges Histogram Equalization (HE) with Contrast-Limited Adaptive Histogram Equalization (CLAHE) to enhance image quality, making low-contrast images easier to see. Additional techniques, including entropy-based analysis and adaptive gamma correction, have been employed to highlight subtle crack details. Goo et al. [18] proposed Hybrid-Segmentor, which integrates CNN and Transformer to enable collaborative feature extraction and demonstrates excellent robustness. Tang et al. [19] introduced VM-UNet++, combining Mamba with the attention mechanism to strike a balance between accuracy and efficiency. Liu et al. [20] developed SCSegamba, featuring a lightweight design suitable for on-site detection.Template-matching methods detect cracks by comparing regions against predefined patterns. Kong Q et al. [21] enhanced this approach by integrating color features, improving the detection of repetitive structural cracks. Beyond traditional image-domain techniques, graph models and energy optimization frameworks have been proposed to ensure edge continuity and regional coherence. Zhou Y [22] is enhancing segmentation accuracy and structural consistency.

With the rapid development of deep learning, CNN-based crack detection methods [23] have increasingly replaced traditional approaches, providing superior accuracy and robustness in complex environments. U-Net, initially developed for medical image segmentation, has proven effective for concrete crack detection due to its encoder-decoder architecture and skip connections [24] and has been widely adopted. Various U-Net variants have been proposed to improve segmentation performance, integrating multi-scale features and attention mechanisms to enhance the detection of fine cracks [25]. In object detection, Faster R-CNN is commonly used to localize cracks via its Region Proposal Network (RPN) [26]. Building on this, Mask R-CNN adds an instance segmentation branch, enabling pixel-level mask output and unifying detection and segmentation. Improved Mask R-CNN models have achieved better accuracy and generalization in bridge crack detection [27]. DeepLabv3 + incorporates Atrous Spatial Pyramid Pooling (ASPP) and a decoder to enhance multi-scale feature extraction. Its attention-based variants improve segmentation performance on concrete and asphalt cracks [28]. Backbone networks are also essential. ResNet addresses gradient vanishing through residual connections and is widely used in crack recognition [29]. DenseNet improves feature propagation with dense connectivity, enhancing segmentation efficiency [30]. VGG networks offer strong feature extraction due to their deep architecture, while EfficientNet balances precision and model size via compound scaling [31,32]. As research advances, transformer-based models have emerged for crack detection due to their global self-attention capabilities. Swin Transformer approaches work well even when they aren't pre-trained [33]. Enhanced Transformer models with multi-scale modules make it easier to segment fine cracks [34]. By integrating a dual encoder with wavelet feature enhancement and multi-stage supervision, this approach addresses the challenges of complex scene segmentation [35,36]. SegFormer, a Transformer-based segmentation model with a hierarchical encoder and lightweight decoder, offers high precision and efficiency. It has outperformed CNN models in detecting fine cracks on concrete and asphalt surfaces [37].

Although deep learning models have made significant progress in crack detection and segmentation, several challenges remain. Many existing networks still struggle to accurately detect fine or fragmented cracks, especially in noisy or low-contrast environments. Architectures such as U-Net and its variations frequently experience information loss during repeated downsampling, causing extremely thin cracks to disappear in deeper layers. Detection-based frameworks like Mask R-CNN [38] may also miss small crack patterns due to anchor constraints. Although transformer-based approaches such as Swin Transformer and SegFormer offer strong global modeling capabilities, they typically require large-scale datasets and substantial computational resources, limiting their practicality in resource-constrained settings. Despite this progress, two fundamental challenges remain insufficiently addressed. First, standard encoder–decoder architectures and lightweight backbones often incur severe feature loss during downsampling, making it difficult to retain low-contrast or hairline cracks. Second, most generic multi-scale and attention mechanisms are originally designed for high-level semantic segmentation and are not specifically adapted to the elongated, noisy, and discontinuous morphology of pavement cracks.

To address these challenges, this paper proposes a novel crack segmentation network, CrackNet, specifically designed for concrete crack recognition. The design of CrackNet draws on principles from information theory, aiming to maximize effective information flow while suppressing noise. CrackNet integrates multi-level feature extraction through three core modules. The LightMSCBlock is introduced into the encoder to capture both local details and global semantics through parallel multi-scale convolutional branches, thereby alleviating the feature loss commonly encountered in traditional convolutional backbones. The SAF attention modules are incorporated into the skip connections to provide crack-oriented recalibration, enhancing weak, fine-scale crack structures while suppressing irrelevant background noise. Finally, the MSFF module is integrated into the decoder to efficiently aggregate multi-scale contextual information with low computational overhead, improving the detection of long, thin, or discontinuous cracks.

## 2 Methodology

**Fig 1:**
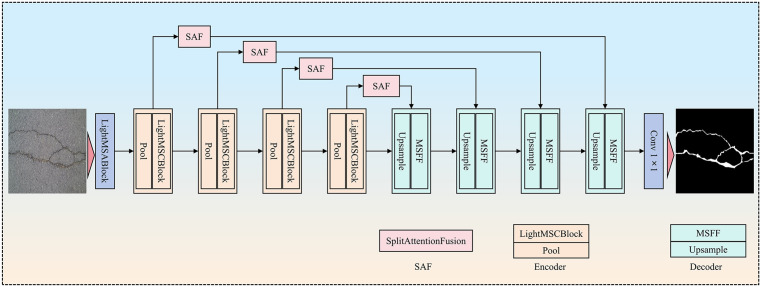
CrackNet architecture for crack segmentation, integrating the LightMSCBlock, MSFF, and SAF modules.

The network architecture proposed in this paper is designed explicitly for crack segmentation. The network has three innovative modules: LightMSCBlock, MSFF, and SAF. LightMSCBlock, the core module of the encoder, extracts multi-scale features from the input image and enhances the representation of important regions via an attention mechanism. The MSFF module, the main component of the decoder, fuses features from different convolutional operations and gradually restores the high-resolution output. The SAF attention module, which is used on skip connections, uses an attention mechanism to improve feature fusion across different scales. This makes sure that details and edge information are transferred correctly. Combining these three innovative modules enables the model to efficiently handle complex images in crack segmentation tasks while delivering high accuracy and robustness. The network architecture is shown in (Fig 1).

Overall, the proposed CrackNet forms a unified encoder–decoder framework in which multi-scale feature extraction, attention-guided feature transmission, and efficient feature fusion are tightly integrated. The LightMSCBlock enhances feature representation at different scales in the encoding stage, the SAF module preserves critical structural details during skip connections, and the MSFF module progressively refines and restores high-resolution features in the decoder. In the following section, we describe the experimental setup, datasets, and evaluation metrics, and present quantitative and qualitative results to validate the effectiveness of the proposed network.

### 2.1 Lightweight Multi-Scale Convolutional Enhancement Block (LightMSCBlock)

**Fig 2:**
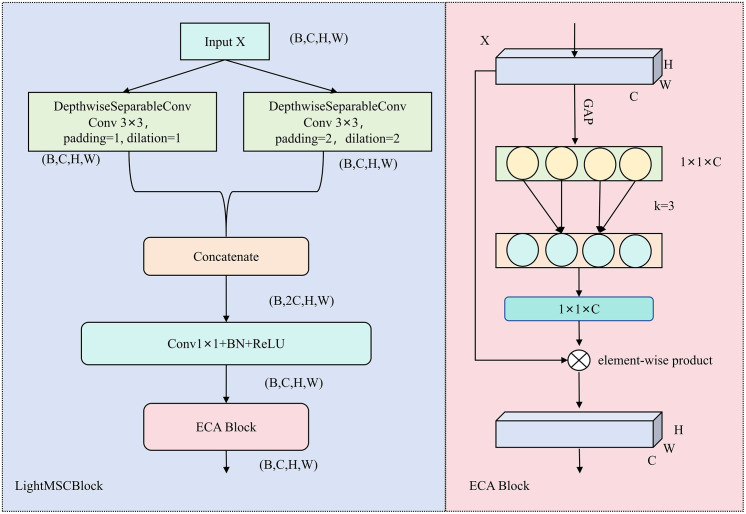
Lightweight Multi-Scale Convolutional Enhancement Block structure diagram.

To simultaneously capture local details and global semantic information, we propose LightMSCBlock (Fig 2). The main design goal of this module is to improve feature representation via multiple convolutions and enhance feature output via attention, thereby improving robustness and generalization across different scenarios.

Given an input feature map $X\in {\mathbb{R}}^{B\times C\times H\times W}$, where *B* denotes the batch size, *C* the number of channels, and *H,W* the spatial resolution.The LightMSCBlock consists of two parallel depthwise separable convolution branches:


$$F_{s}=\varphi_{ds}(X;k=3,d=1),F_{d}=\varphi_{ds}(X;k=3,d=2)$$ (1)

where $\varphi_{ds}(.)$ represents depthwise separable convolution, k is the kernel size, and d is the dilation rate. The standard branch $F_{s}$ focuses on fine-grained local features, whereas the dilated branch $F_{d}$ enlarges the receptive field to capture broader contextual information. This parallel design enables the module to simultaneously integrate local and global cues, providing discriminative representations for downstream tasks.

The outputs of both branches are concatenated and fused via a 1 × 1 convolution followed by batch normalization and a ReLU activation:


$$F=\sigma \left(BN\left(W_{1\times 1}.[F_{s};F_{d}]\right)\right)$$ (2)

where $\left[F_{s};F_{d}\right]$ denotes concatenation, *W*_*1*_ *× W*_*1*_ is the fusion kernel, BN denotes batch normalization, and σ(.) is the ReLU function. This operation not only integrates multi-scale information but also suppresses redundancy and alleviates the computational burden associated with traditional multi-branch structures.

To further improve the informative features, an Efficient Channel Attention (ECA) [39] mechanism is integrated after the fusion. Specifically, channel descriptors are derived through global average pooling:


$$z_{c}=\frac{\text{1}}{H\times W}\sum_{i=1}^{H}\sum_{j=1}^{W}F_{c}\left(i,j\right),z\in {\mathbb{R}}^{C}$$ (3)

Where $F_{c}\left(i,j\right)$ denotes the activation of the c-th channel at position (i,j). A lightweight 1D convolution is then applied to model inter-channel dependencies:


$$s=\sigma \left(Conv1D\left(z;k\right)\right),s\in {\mathbb{R}}^{C}$$ (4)

with kernel size *k* and Sigmoid activation σ. Finally, the recalibrated feature is obtained by channel-wise multiplication:


$$F^{\prime }=F⊗s$$ (5)

where ⨂ denotes channel-wise multiplication.

LightMSCBlock seamlessly integrates lightweight computation, explicit multi-scale modeling, and efficient feature enhancement. Unlike traditional multi-scale convolutional architectures that introduce high computational cost, LightMSCBlock preserves sufficient representational power while extracting features at multiple receptive fields within a single block. In addition, compared to standard SE-style channel recalibration, the incorporation of ECA avoids information loss caused by dimensionality reduction and provides more flexible and accurate channel modeling. Beyond these general advantages, LightMSCBlock also differs fundamentally from the depthwise-separable inverted residual blocks in MobileNet and EfficientNet. First, MobileNet/EfficientNet capture multi-scale information implicitly through stacking blocks at different depths, whereas LightMSCBlock introduces explicit parallel multi-scale branches within a single block, enabling receptive-field aggregation before any downsampling takes place. Second, while EfficientNet applies squeeze-and-excitation to a single fused feature map, LightMSCBlock performs joint multi-scale feature fusion combined with lightweight attention, which better preserves the fine, low-contrast crack structures that are easily lost during downsampling. These characteristics make LightMSCBlock particularly suitable for crack detection tasks, where thin and subtle patterns require careful preservation of detailed information.

**Algorithm 1.** **Lightweight Multi-Scale Convolutional Enhancement Block.**

**Input:** Feature map $X\in {\mathbb{R}}^{B\times C\times H\times W}$.

**Output:** Enhanced feature map $F^{\prime }\in {\mathbb{R}}^{B\times C\times H\times W}$.

1: Initialize standard depthwise separable branch *φ*_*ds*_(‧;*k*=3, *d*=1).

2: Initialize dilated depthwise separable branch *φ*_*ds*_(‧;*k*=3, *d*=2).

3: Initialize fusion module with 1 × 1 convolution *W*_*1 × 1*_, batch normalization BN, and ReLU activation *σ(·)*.

4: Initialize Efficient Channel Attention (ECA) module with global average pooling, 1D convolution (kernel size *k*), and Sigmoid activation.

5: Given input feature map *X*∈ℝ^*B*×*C*×*H*×*W*^.

6: Compute the standard branch output using Eq. (1):

7:  $F_{s}=\phi_{ds}(X;k=3,d=1)$;

8: Compute the dilated branch output using Eq. (1):

9:  $F_{d}=\phi_{ds}(X;k=3,d=2)$;

10: Concatenate the outputs of the two branches along the channel dimension:

11:  *F*_*cat*_ *= [Fs; Fd]*;

12: Fuse multi-scale features using Eq. (2):

13:  *F*=*σ*(*BN*(*W*_1 × 1_ [*F*_*s*_;*F*_*d*_]));

14: Apply global average pooling on F to obtain channel descriptors using Eq. (3):

15:  **for** each channel *c* = 1 to *C*_*out*_ do

16:   $z_{c}=\frac{\text{1}}{H\times W}\sum_{i=1}^{H}\sum_{j=1}^{W}F_{c}(i,j),z\epsilon {\mathbb{R}}^{C}$;

17:  **end**;

18: Model inter-channel dependencies with a 1D convolution and Sigmoid activation using Eq. (4):

19:  *s*=*σ*(*Conv*1*D*(*z*;*k*)), *s*∈ℝ^c^;

20: Reshape and broadcast s to match the dimensions of *F*.

21: Recalibrate the fused feature map by channel-wise multiplication using Eq. (5):

22:  $F^{\prime }=F⨂s$;

23: **return** $the\;enhanced\;feature\;map\;F^{\prime }$;

### 2.2 SAF attention module

**Fig 3:**
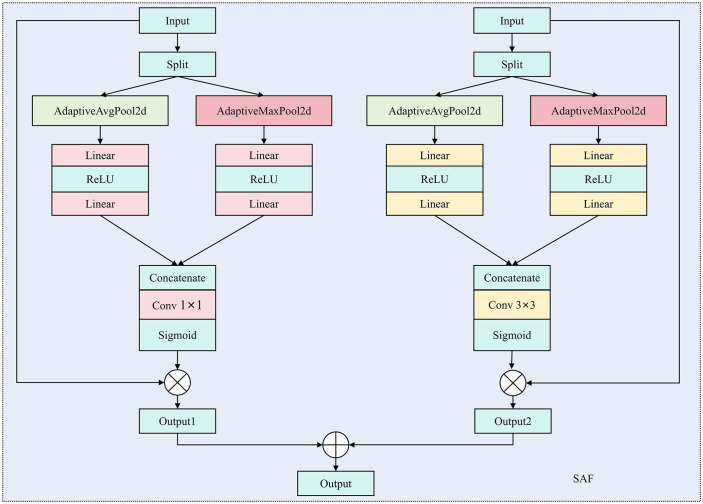
SAF attention structure diagram.

In this work, we propose the SAF attention module (Fig 3), a novel channel attention mechanism that enhances feature representation by efficiently combining multi-scale information. This module leverages adaptive average pooling and adaptive max pooling to extract features at different scales, followed by 1 × 1 and 3 × 3 convolutions to generate attention weights for each channel. Combining these operations allows the model to focus on the most relevant features while maintaining computational efficiency.

Given an input feature map $X\in {\mathbb{R}}^{B\times C\times H\times W}$, where *B* represents the batch size, *C* the number of channels, and *H* and *W* the spatial dimensions, the input is first split along the channel dimension:


$$X_{\text{1}},X_{\text{2}}=torch.split(X,\frac{C}{\text{2}},dim=1)$$ (6)

where $X_{\text{1}}$ and $X_{\text{2}}$ each contain $\frac{C}{\text{2}}$ channels. The first branch applies adaptive average pooling to $X_{\text{1}}$, while the second branch applies adaptive max pooling to $X_{\text{2}}$:


$$avg\_out=avgpool\left(X_{\text{1}}\right)\;,\;max\_out=max\;pool\left(X_{\text{2}}\right)$$ (7)

where avgpool and maxpool reduce the spatial dimensions of each branch to *1 × 1*. These pooled outputs are then passed through fully connected layers for dimensionality reduction and recovery:


$$avgpool=FC_{avg}(avg\_out),max\;pool=FC_{max}(max\_out)$$ (8)

where *FC*_*avg*_ and *FC*_*max*_ are composed of two linear layers (for reduction and recovery) and ReLU activations.

The outputs from both branches are concatenated:


merged=[avgout,max out] (9)

and passed through two separate convolutions 1 × 1 convolution and 3 × 3 convolution-to generate attention maps for different scales:


$$att_{11}=\left(Conv_{1\times 1}\left(merged\right)\right),att_{33}=\sigma \left(Conv_{3\times 3}\left(merged\right)\right)$$ (10)

where σ(.) is the Sigmoid activation function, and conv1x1 and conv3x3 are the convolutional layers used to generate the attention weights at different scales. The final attention map is obtained by combining the two attention maps:


$$X^{\prime }=X⊗(att_{11}+att_{33})$$ (11)

where ⨂ denotes element-wise multiplication, which applies the attention weights to the input feature map.

Unlike the traditional Squeeze-and-Excitation (SE) [40] module, the proposed SAF attention removes the dimensionality-reduction step during channel recalibration, thereby avoiding the loss of discriminative information. In SE, the use of fully connected layers for channel compression may discard critical responses, particularly when the feature maps are high-dimensional. SAF instead captures channel dependencies through multi-scale pooling combined with a convolution-based attention generator, preserving richer information and enabling more flexible and fine-grained channel modelling, which leads to improved performance. Beyond this advantage, our SAF modules are conceptually related to existing attention mechanisms such as CBAM and ECA, yet they are specifically designed for the characteristics of pavement cracks. CBAM applies channel and spatial attention sequentially and employs relatively heavy 2D pooling operations, increasing computational overhead while not being optimized for elongated, thin crack structures. ECA, which we also include as an internal baseline, performs only channel-wise modelling without incorporating spatial cues, making it less effective for capturing the fine geometry of cracks. In contrast, SAF introduce lightweight spatial attention with multi-scale pooling and joint feature weighting, enabling the network to selectively enhance long, thin, and discontinuous crack patterns while suppressing background noise. This design achieves a more favorable balance between accuracy and efficiency for crack detection compared to generic attention modules.

**Algorithm 2.** **SAF Attention Module.**

**Input:** Feature map $X\in {\mathbb{R}}^{B\times C\times H\times W}$.

**Output:** Refined feature map $X^{\prime }\in {\mathbb{R}}^{B\times C\times H\times W}$.

1: Set split_channels = C/2.

2: Split the input feature map along the channel dimension using Eq. (6):

3:  *X*_*1*_, *X*_*2*_ = *split(X, split_channels, dim = 1);*

4: Apply adaptive average pooling and adaptive max pooling using Eq. (7):

5:  *avg_out = avgpool(X*_*1*_), *max_out = maxpool(X*_*2*_);

6: Pass pooled features through branch-specific fully connected layers with reduction and recovery using Eq. (8):

7:  *avg_out = FC_avg(avg_out), max_out = FC_max(max_out)*;

8: Concatenate the two branch outputs along the channel dimension using Eq. (9):

9:   *merged = [avg_out; max_out]*;

10: Generate multi-scale attention maps with 1 × 1 and 3 × 3 convolutions using Eq. (10):

11:   *att*_*11*_ *= Conv*_*1x1*_*(merged), att*_*33*_ *= Sigmoid(Conv*_*3x3*_*(merged))*;

12:  where Sigmoid(·) denotes the Sigmoid activation function.

13: Combine the attention maps and recalibrate the input feature map using Eq. (11):

14:  $X^{\prime }=X⊗\left(att_{11}+att_{33}\right)$;

15:  where ʘ denotes element-wise multiplication.

16: **return** $the\;refined\;feature\;map\;X^{\prime }$;

### 2.3 Multi-Scale Feature Fusion Module (MSFF)

**Fig 4:**
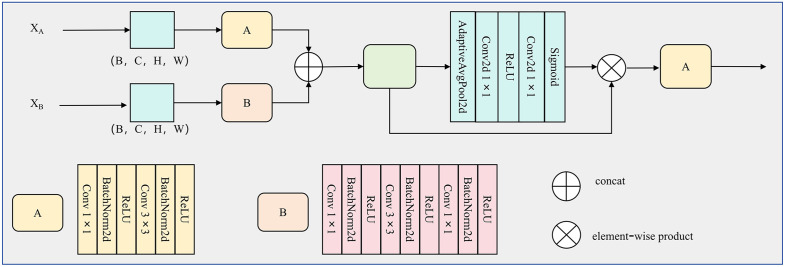
Multi-Scale Feature Fusion Module diagram.


To explicitly model channel-wise dependencies in the fused multi‑scale features, we integrate a Squeeze‑and‑Excitation (SE)-based channel attention mechanism into the MSFF module (Fig 4). Given the outputs of the two convolutional branches, $X_{A},X_{B}\in {\mathbb{R}}^{B\times C\times H\times W}$, we first fuse them along the channel dimension to preserve the information from different scales:

$$X_{f}=Concat\left(X_{A},X_{B}\right),\text{\textbackslash{}hspace\{0.17em}\}X_{A},X_{B}\in {\mathbb{R}}^{B\times C\times H\times W}$$ (12)

On top of *X*_*f*_, the SE block performs a squeeze-excitation operation to produce channel attention weights. In the squeeze step, global average pooling is applied to aggregate spatial information of each channel into a scalar descriptor:


$$z_{c}=\frac{\text{1}}{HW}\sum_{i=1}^{H}\sum_{j=1}^{W}\overset{\left(c\right)}{\underset{f}{X}}(i,j),\;c=1,...,2C$$ (13)

which yields $z\in {\mathbb{R}}^{B\times 2C\times 1\times 1}$. In the excitation step, *z* is passed through two successive 1 × 1 convolutions (equivalent to fully connected layers on the channel dimension) with a reduction ratio r, followed by ReLU and Sigmoid activations:


$$s=\sigma (W_{\text{2}}\delta (W_{\text{1}}z))$$ (14)

where $W_{\text{1}}\in {\mathbb{R}}^{\frac{2C}{r}\times 2C}$ and $W_{\text{2}}\in {\mathbb{R}}^{2C\times \frac{2C}{r}}$ are the learnable weights of the two 1 × 1 convolutions, δ denotes the ReLU function, and σ is the Sigmoid function. The resulting vector $s\in {\mathbb{R}}^{B\times 2C\times 1\times 1}$ encodes the importance of each channel. Finally, the attention weights are broadcast and applied to the fused feature map via channel‑wise multiplication:


$${\tilde{X}}_{f}=X_{f}⨀s$$ (15)

where ⊙ denotes element‑wise multiplication. The reweighted feature ${\tilde{X}}_{f}$ is then fed into the subsequent hybrid convolution layer to generate the final output of the MSFF module. In this way, the proposed channel attention mechanism adaptively emphasizes informative channels from different scales while suppressing redundant or irrelevant responses, leading to a more compact and discriminative multi‑scale representation. Unlike traditional multi-scale fusion strategies that rely on repeatedly stacking convolutions or employing multiple scale-specific kernels, the MSFF module avoids redundant computation. By efficiently combining 1 × 1 and 3 × 3 convolutions with channel attention, MSFF extracts more precise multi-scale features while maintaining low computational overhead, offering a more efficient alternative to conventional multi-layer or multi-branch fusion designs. The proposed MSFF is conceptually related to the atrous spatial pyramid pooling (ASPP) module in DeepLab, as both aim to aggregate contextual information at multiple scales. However, ASPP employs several parallel dilated convolutions with large dilation rates, significantly increasing computational cost and potentially introducing gridding artifacts. Moreover, ASPP is designed for high-level semantic segmentation, whereas crack detection demands the preservation of fine, low-level geometric structures. In contrast, MSFF integrates lightweight multi-scale pooling and convolution with attention-based adaptive weighting of scale-specific features. This design not only reduces computation but also preserves local crack continuity more effectively, making the module particularly suitable for deployment in resource-constrained crack detection scenarios.

## 3 Experiments and results

### 3.1 Datasets

**Fig 5:**
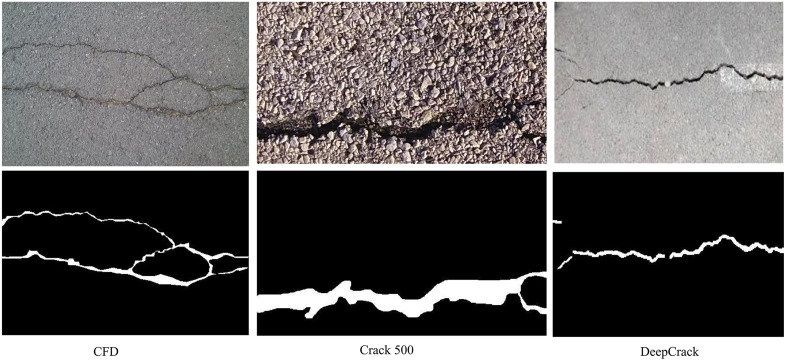
Dataset visualization (original images and label images of three types of data sets).

In this study, three datasets were used for model training: the CFD [41] dataset, the Crack500 [42] dataset, and the DeepCrack [43] dataset (Fig 5). The publicly available CFD dataset contains 118 crack images with a resolution of 480 × 320 pixels, which include noise such as water stains and shadows. The Crack500 dataset comprises 500 high-resolution images (2000 × 1500 pixels) of real road cracks. It features high scene complexity, including various crack types, diverse background materials, and lighting variations, making it suitable for evaluating model robustness in complex environments. The DeepCrack dataset comprises 591 images from multiple crack image collections, with an original resolution of 544 × 544 pixels. It includes a wide range of crack patterns and background noise, such as dust, scratches, and lighting interference, and is commonly used for training deep crack detection models.

Due to the relatively small size of the three datasets and the proposed model's requirement for input images of 512 × 512 pixels, data augmentation techniques were applied. These techniques included random cropping, brightness adjustment, contrast adjustment, and rotation. As a result, the CFD dataset was expanded to 2,000 images, the Crack500 dataset to 3,600 images, and the DeepCrack dataset to 3,000 images. Each dataset was first split into training, validation, and test sets in a ratio of 8:1:1. After the data split, augmentation was applied only to the training set, while the validation and test sets were resized deterministically to maintain their original content. This process ensures that no augmented images from the training set overlap with the validation or test sets, avoiding any potential data leakage.

### 3.2 Experimental setup and evaluation index

The proposed model was developed using Python 3.10 and built on the open-source deep learning framework PyTorch. The training process was accelerated using CUDA 12.6. The hardware environment consisted of an NVIDIA RTX 4090 GPU with 24 GB of VRAM. During training, the Adam optimizer was employed. The model was trained for 50 epochs with a batch size of 8 and an initial learning rate of 0.001. An early stopping mechanism was applied during training. Precision is defined as the ratio of correctly predicted positive samples to the total number of predicted positive samples:


$$precision=\frac{TP}{TP+FP}$$ (16)

The recall rate is calculated as the proportion of all actual targets correctly predicted:


$$recall=\frac{TP}{TP+FN}$$ (17)

Among them, TP is the number of targets correctly detected, FP is the number of targets incorrectly detected, and FN is the number of targets missed in the actual correct targets.

The F1 score combines precision and recall, providing a more comprehensive measure of the network's overall performance. It is calculated as a harmonic mean of these two metrics, as shown below:


$$F1=\frac{2*precision*recall}{precision+recall}$$ (18)

The standard definition of IoU is to calculate the ratio of the overlapping area to the union area between two regions (usually bounding boxes), as follows:


$$IoU=\frac{TP}{TP+FP+FN}$$ (19)

### 3.3 Loss function

To train the proposed model for crack detection, we employ a combined loss function that integrates both Cross-Entropy Loss and Dice Loss. The Cross-Entropy Loss is used for pixel-wise classification, encouraging the model to distinguish between crack and background pixels. The formula for Cross-Entropy Loss is given by:


$$Cross-Entropy\;Loss=-\sum_{i=1}^{N}\left[y_{i}log(p_{i})+(1-y_{i})log(1-p_{i})\right]$$ (20)

Where $y_{i}$ represents the ground truth label, $p_{i}$ is the predicted probability, and *N* is the number of pixels. Since cracks often occupy a small portion of the image, class imbalance is a significant issue. To address this, Dice Loss is introduced, which enhances the model’s ability to detect thin cracks by measuring the overlap between the predicted and ground truth masks. The Dice Coefficient is defined as:


$$Dice\;Coefficient=\frac{2\sum_{i=1}^{N}y_{i}p_{i}}{\sum_{i=1}^{N}y_{i}+\sum_{i=1}^{N}p_{i}}$$ (21)

The Dice Loss is then computed as:


Dice Loss=1−Dice Coefficient (22)

The final loss function is the weighted sum of Cross-Entropy Loss and Dice Loss:


Total Loss=Cross−Entropy Loss+λ·Dice Loss (23)

Where λ is a hyperparameter controlling the relative weight of the Dice Loss. In our experiments, we set λ = 1, giving equal importance to both losses. This combined approach allows the model to balance pixel-wise accuracy with the preservation of fine, low-contrast crack structures.

### 3.4 Experimental results and analysis

#### 3.4.1 Ablation experiment.

We conducted ablation experiments on the CFD dataset to evaluate the contribution of each proposed module (Table 1). The base model, a standard U-Net architecture, achieved a precision of 0.7167, recall of 0.7258, F1 score of 0.7213, and IoU of 0.5641. Introducing the LightMSCBlock (A) improved multi-scale feature extraction, while the SAF attention module (B) enhanced feature discrimination by suppressing background noise. The MSFF module (C) further strengthened multi-scale contextual aggregation during decoding. Each module individually offered performance gains compared to the base model.

**Table 1:** Ablation experiments of the network on CFD dataset.

model	Pr	recall	F1	Iou
Base	0.7167	0.7258	0.7213	0.5641
Base+A	0.7233	0.7471	0.7201	0.5741
Base+B	0.7196	0.7323	0.7243	0.5512
Base+C	0.7172	0.7412	0.7256	0.5638
Base+A + B	0.7285	0.7561	0.7289	0.5987
Base+A + C	0.7243	0.7689	0.7358	0.5941
Base+B + C	0.7266	0.7946	0.7413	0.5987
Base+A + B + C	**0.7325**	**0.8128**	**0.7550**	**0.6064**

We also evaluated pairwise combinations of the modules. The A + C configuration improved recall and F1 by enhancing both encoder-level multi-scale representation and decoder-level contextual fusion. The B + C combination further boosted recall (0.7946) and F1 (0.7413), demonstrating that attention-guided feature recalibration (B) and multi-scale fusion (C) complement each other effectively. When all three modules were integrated (A + B + C), the full model achieved the best performance, yielding a precision of 0.7325, recall of 0.8128, F1 score of 0.7550, and IoU of 0.6064. This substantial improvement indicates that the three modules act synergistically: LightMSCBlock preserves fine crack details during downsampling, SAF enhances crack-relevant spatial-channel responses in skip connections, and MSFF improves structural continuity during upsampling. Overall, the results demonstrate that each module contributes to segmentation accuracy from different stages of the network pipeline, and their combination yields robust and significant performance gains, confirming the effectiveness of the proposed CrackNet architecture for crack segmentation.

Table 2 summarizes the ablation results on the Crack500 dataset. The baseline U-Net model achieved a precision of 0.6576, recall of 0.7542, F1 score of 0.7026, and IoU of 0.5416. Introducing the LightMSCBlock (A) yielded consistent improvements across all metrics, demonstrating its ability to enhance multi-scale feature representation. Incorporating the SAF attention module (B) slightly decreased precision to 0.6543 but improved recall and F1, indicating that the attention mechanism strengthens crack-focused feature responses while marginally increasing false positives. The MSFF module (C) further improved recall and F1 while maintaining stable precision, reflecting enhanced multi-scale contextual aggregation in the decoder. Pairwise combinations of the modules produced additional gains. The A + C configuration achieved higher recall (0.7851) and F1 (0.7546), showing the complementary effect of encoder-level multi-scale enhancement and decoder-level fusion. The B + C combination yielded further improvement, with an IoU of 0.6104, confirming that attention-guided recalibration (B) and multi-scale feature fusion (C) jointly strengthen crack continuity and structural consistency. When all three modules were integrated (A + B + C), the model achieved the highest performance across all metrics, with a notable increase in precision to 0.8765 and stable recall at 0.7964. This substantial precision increase can be attributed to the synergistic effect of the three modules: LightMSCBlock reduces information loss during downsampling, SAF suppresses background noise and enhances discriminative crack responses, and MSFF improves multi-scale context aggregation. Together, these mechanisms significantly reduce false positives while maintaining strong crack detection capability, resulting in improved F1 (0.7636) and IoU (0.6175).

**Table 2:** Ablation experiments of network on Crack500 dataset.

model	Pr	recall	F1	Iou
Base	0.6576	0.7542	0.7026	0.5416
Base+A	0.6579	0.7643	0.7431	0.5712
Base+B	0.6543	0.7586	0.7325	0.5645
Base+C	0.6589	0.7557	0.7423	0.5632
Base+A + B	0.6601	0.7754	0.7504	0.5986
Base+A + C	0.6984	0.7851	0.7546	0.6013
Base+B + C	0.7578	0.7896	0.7612	0.6104
Base+A + B + C	**0.8765**	**0.7964**	**0.7636**	**0.6175**

Table 3 presents the ablation results of different module combinations on the DeepCrack dataset. The baseline U-Net model achieved a precision of 0.7014, recall of 0.7965, F1 score of 0.7459, and IoU of 0.5948. When incorporating the LightMSCBlock (A), performance improved across all metrics, reaching an F1 of 0.7944 and IoU of 0.6476, indicating that encoder-level multi-scale representation substantially enhances feature retention and crack boundary detection. Adding the SAF module (B) also improved overall performance, achieving an F1 of 0.7856 and IoU of 0.6561. This result suggests that attention-based feature recalibration strengthens crack saliency while maintaining robust spatial segmentation. The MSFF module (C) provided additional benefits in multi-scale fusion, increasing IoU to 0.6778 and yielding more consistent segmentation outputs across varying crack widths and textures. Pairwise module combinations further validate the complementary nature of these components. The A + B configuration increased F1 to 0.8131 and IoU to 0.7070, demonstrating the synergy between multi-scale enhancement and attention-guided feature refinement. The A + C combination performed even better, with an F1 of 0.8294 and IoU of 0.7156, reflecting improved encoder–decoder coupling through stronger multi-scale fusion. The B + C combination achieved one of the highest pairwise results, with an F1 of 0.8369 and IoU of 0.7196, confirming that the combination of attention and multi-scale fusion effectively improves crack continuity and reduces misclassification. When all three modules were integrated (A + B + C), the model achieved the best performance: 0.8654 precision, 0.8625 recall, 0.8423 F1, and 0.7275 IoU. This demonstrates that the three modules address complementary aspects of crack segmentation—A enhances fine feature retention, B improves crack-focused recalibration, and C strengthens multi-scale integrative reasoning—resulting in a robust and comprehensive performance improvement. Overall, the ablation results confirm that each module contributes meaningfully to segmentation quality, and their full integration yields the most accurate, consistent, and reliable detection performance on the DeepCrack dataset.

**Table 3:** Ablation experiments of network on DeepCrack dataset.

model	Pr	recall	F1	Iou
Base	0.7014	0.7965	0.7459	0.5948
Base+A	0.7687	0.8021	0.7944	0.6476
Base+B	0.7546	0.7945	0.7856	0.6561
Base+C	0.7478	0.7906	0.7636	0.6778
Base+A + B	0.8346	0.8107	0.8131	0.7070
Base+A + C	0.8446	0.8303	0.8294	0.7156
Base+B + C	0.8512	0.8498	0.8369	0.7196
Base+A + B + C	**0.8654**	**0.8625**	**0.8423**	**0.7275**

#### 3.4.2 Comparison experiment.

To comprehensively evaluate the performance of different semantic segmentation models in crack detection tasks, we selected several mainstream architectures, including Unet, VGG16UNet, MobileV3Unet, SegFormer, FCN_ResNet50, and our proposed model, Ours. Comparative experiments were conducted on three publicly available crack datasets: CFD, Crack500, and DeepCrack. We adopted four quantitative metrics to ensure objective and thorough evaluation: precision, recall, F1, and intersection over union (IoU). Through these experiments, we aim to validate each model’s strengths and its applicability to crack detection.

**Fig 6:**
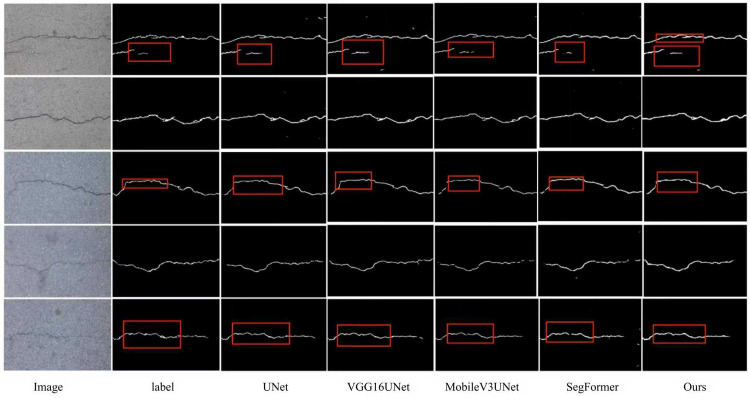
Visualization of the model compared with other models on the CFD dataset.

Table 4 presents a comparison of various models on the CFD dataset, evaluated using Pr, recall, F1, and IoU. The baseline UNet model achieves a precision of 0.7167, a recall of 0.7258, an F1 of 0.7213, and an IoU of 0.5641, serving as the benchmark for comparison. VGG16UNet shows a slight decrease in precision (0.6793) but achieves higher recall (0.7538) than UNet, resulting in a slightly lower F1 (0.7147) and a slight reduction in IoU (0.556). This indicates that while VGG16UNet improves recall, it sacrifices some precision and spatial consistency. MobileV3Unet excels in precision (0.7529) but suffers from a significantly lower recall (0.6208) and F1 (0.6805), with a considerably lower IoU (0.5157). This suggests that while MobileV3Unet can correctly classify positives, it fails to capture many true positives, severely impacting its segmentation performance. SegFormer achieves a moderate Pr (0.6433) and recall (0.7471), with an F1 of 0.6913 and IoU of 0.5352, indicating decent recall performance but lower precision and overall segmentation accuracy. FCN_resnet50 shows the lowest precision (0.5775) but a high recall (0.7617), resulting in the lowest F1 (0.6570) and IoU (0.4891), suggesting that while fcn_resnet50 excels at detecting true positives, it struggles with accurate segmentation boundaries. Finally, ours outperforms all other models, achieving a precision of 0.7325, a recall of 0.8128, an F1 of 0.7550, and an IoU of 0.6064, demonstrating the best balance between classification accuracy and spatial consistency. In summary, while other models may excel in one or two metrics, ours provides a more well-rounded performance, achieving superior results across all evaluated metrics, making it the most effective model for the CFD dataset. Fig 6 shows a visual comparison of the model with other models on the CFD dataset.

**Table 4:** Comparison test of network on CFD dataset.

model	pr	recall	F1	Iou
Unet	0.7167	0.7258	0.7213	0.5641
VGG16UNet	0.6793	0.7538	0.7147	0.5560
ResUNet	0.6743	0.7689	0.6911	0.5497
AttentionUNet	0.6987	0.7846	0.7102	0.5649
MobileV3Unet	0.7529	0.6208	0.6805	0.5157
SegFormer	0.6433	0.7471	0.6913	0.5352
fcn_resnet50	0.5775	0.7617	0.6570	0.4891
DeepCrack	0.7471	0.7956	0.7311	0.5945
OUrs	**0.7325**	**0.8128**	**0.7550**	**0.6064**

**Fig 7:**
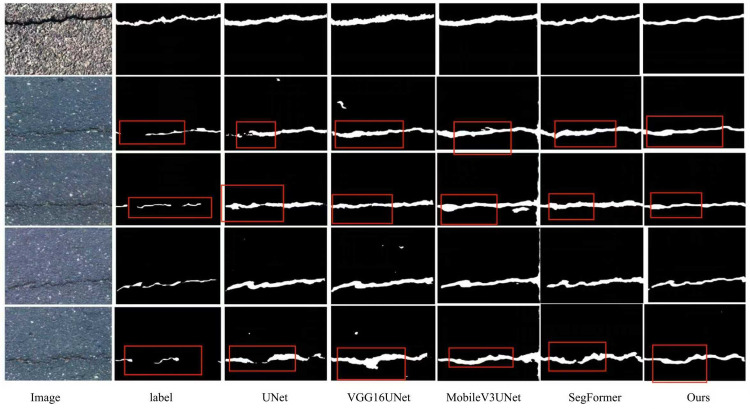
Visualization of the model compared with other models on the Crack500 dataset.

Table 5 compares various models on the Crack500 dataset, evaluated using Pr, recall, F1, and IoU. The baseline UNet model achieves a precision of 0.6576, recall of 0.7542, an F1 of 0.7026, and an IoU of 0.5416, providing a solid reference for comparison. VGG16UNet achieves a precision of 0.6807, a recall of 0.7067, an F1 of 0.6934, and an IoU of 0.5307, showing improved precision but lower recall and IoU compared to UNet, indicating that while VGG16UNet improves precision, it sacrifices recall and spatial consistency. MobileV3Unet shows a precision of 0.6966, a recall of 0.7559, an F1 of 0.725, and an IoU of 0.5687, demonstrating an improvement in recall and F1 over VGG16UNet, with a higher IoU than both VGG16UNet and UNet, suggesting better overall performance in segmentation tasks. SegFormer achieves a precision of 0.8334, a recall of 0.6529, an F1 of 0.7322, and an IoU of 0.5776, which shows the highest precision among all models tested, but at the cost of significantly lower recall, resulting in an imbalance in classification and recall. fcn_resnet50 achieves a precision of 0.8236, a recall of 0.6839, an F1 of 0.7473, and an IoU of 0.5966, showing strong performance in precision and recall, with relatively high F1 and IoU, but still slightly behind MobileV3Unet and Ours in terms of overall segmentation accuracy. Finally, Ours outperforms all other models, achieving a precision of 0.8765, a recall of 0.7964, an F1 of 0.7636, and an IoU of 0.6175, demonstrating the best balance among precision, recall, and spatial accuracy. In summary, Ours achieves the best overall performance on the Crack500 dataset, with significant improvements in recall, F1, and IoU, outperforming all other models across all evaluated metrics, highlighting its effectiveness in segmentation tasks. Fig 7 visually compares the model with other models on the Crack500 dataset.

**Table 5:** Comparison test of network on Crack500 dataset.

model	pr	recall	F1	Iou
Unet	0.6576	0.7542	0.7026	0.5416
VGG16UNet	0.6807	0.7067	0.6934	0.5707
ResUNet	0.6821	0.7164	0.7243	0.5369
AttentionUNet	0.6832	0.7236	0.7169	0.5443
MobileV3Unet	0.6966	0.7559	0.7250	0.5687
SegFormer	0.8334	0.6529	0.7322	0.5776
Fcn_resnet50	0.8236	0.6839	0.7473	0.5966
DeepCrack	0.8463	0.7489	0.7529	0.6111
OUrs	**0.8765**	**0.7964**	**0.7636**	**0.6175**

**Fig 8:**
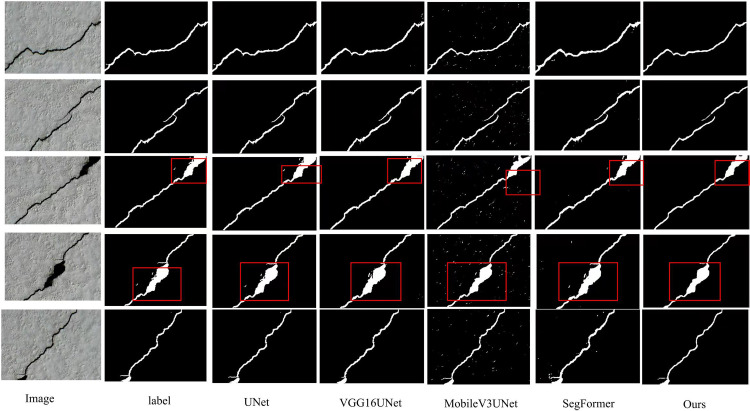
Visualization of the model compared with other models on the DeepCrack dataset.

Table 6 compares DeepCrack models evaluated using Pr, recall, F1, and IoU. The baseline UNet model achieves precision of 0.7014, recall of 0.7965, F1 of 0.7459, and IoU of 0.5948, serving as the reference point for comparison. VGG16UNet demonstrates significant improvements, achieving a precision of 0.8324, a recall of 0.8293, an F1 of 0.8156, and an IoU of 0.6652, outperforming UNet in classification and recall. MobileV3Unet achieves precision of 0.8236, recall of 0.8454, F1 of 0.7856, and IoU of 0.7021, outperforming both VGG16UNet and UNet in recall and IoU, indicating a better balance between precision and spatial accuracy. SegFormer shows a precision of 0.7602, recall of 0.8459, F1 of 0.8008, and IoU of 0.6678, performing similarly to MobileV3Unet in recall but with slightly lower precision and IoU. fcn_resnet50 achieves a precision of 0.8312, a recall of 0.7623, an F1 of 0.8049, and an IoU of 0.6735, showing strong performance in precision and F1, but lower recall than MobileV3Unet. Finally, Ours outperforms all other models, achieving a precision of 0.8654, a recall of 0.8625, an F1 of 0.8423, and an IoU of 0.7275, demonstrating the best balance among precision, recall, and spatial accuracy. In summary, Ours performs best on the DeepCrack dataset, surpassing all other models across all metrics, highlighting its superior ability to achieve high classification accuracy and precise segmentation. Fig 8 visually compares the model with other models on the DeepCrack dataset.

**Table 6:** Comparison test of network on DeepCrack dataset.

model	pr	recall	F1	Iou
Unet	0.7014	0.7965	0.7459	0.5948
VGG16UNet	0.8324	0.8293	0.8156	0.6652
ResUNet	0.8354	0.8348	0.8179	0.6541
AttentionUNet	0.8226	0.8354	0.8263	0.6897
MobileV3Unet	0.8236	0.8454	0.7856	0.7021
SegFormer	0.7602	0.8459	0.8008	0.6678
fcn_resnet50	0.8312	0.7623	0.8049	0.6735
DeepCrack	0.8346	0.8515	0.8215	0.7112
OUrs	**0.8654**	**0.8625**	**0.8423**	**0.7275**

#### 3.4.3 Comparative experiment between SAF attention and other attention.

Table 7 summarizes the comparative results of different attention mechanisms and the proposed SAF attention on three crack segmentation datasets. The CFD dataset’s SE, CAM, and CE performances were relatively close. In contrast, SAF attention produced a slightly higher F1 (0.755) than the others, suggesting a more favorable balance between precision and recall. For the DeepCrack dataset, SAF attention achieved an accuracy of 0.8654, marginally higher than the alternative mechanisms, with a recall similar to theirs, leading to the highest F1 (0.8214) among all methods. On the Crack500 dataset, SAF attention again achieved better values, with both precision (0.8765) and recall (0.7964) higher than those of the other mechanisms, resulting in an F1 of 0.7636, compared to approximately 0.753 for SE, CAM, and CE. Overall, these findings indicate that although differences across methods are not always significant, SAF attention tends to yield more consistent improvements in segmentation performance, particularly on datasets with diverse crack scales and complex backgrounds, thereby enhancing the model's robustness under varying conditions.

**Table 7:** Comparative experiments between different attention and SAF attention on three datasets.

	CFD			DeepCrack			Crack500		
Method	Pr	Recall	F1	Pr	Recall	F1	Pr	Recall	F1
with/SE	0.7301	0.8111	0.7356	0.8538	0.8056	0.8203	0.8590	0.7854	0.7531
with/CAM	0.7321	0.8124	0.7421	0.8524	0.8123	0.8208	0.8474	0.7824	0.7534
with/CE	0.7213	0.8107	0.7422	0.8540	0.8111	0.8178	0.8452	0.7825	0.7521
with/SAF	**0.7325**	**0.8128**	**0.755**	**0.8654**	**0.8126**	**0.8214**	**0.8765**	**0.7964**	**0.7636**

**Fig 9:**
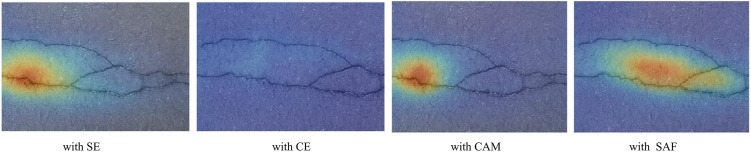
Visualization of attention responses on a sample crack image using different mechanisms. From left to right and top to bottom: SE, CAM, CE, and SAF. The heatmaps highlight the regions where the model focuses during crack segmentation.

Fig 9 illustrates the attention visualization results of different mechanisms on a representative crack image. The SE and CAM modules emphasize localized regions, capturing only parts of the crack and occasionally overlooking its continuity. CE produces a more dispersed activation that covers broader areas but lacks precise alignment with the crack boundaries. In contrast, the SAF attention provides a more coherent, continuous focus across the entire crack, effectively highlighting both the central structure and its extensions. These qualitative observations are consistent with the quantitative improvements reported in Table 7, suggesting that incorporating SAF attention enhances the model’s ability to capture long-range dependencies and represent crack structures more completely.

#### 3.4.4 Model complexity comparison.

**Fig 10:**
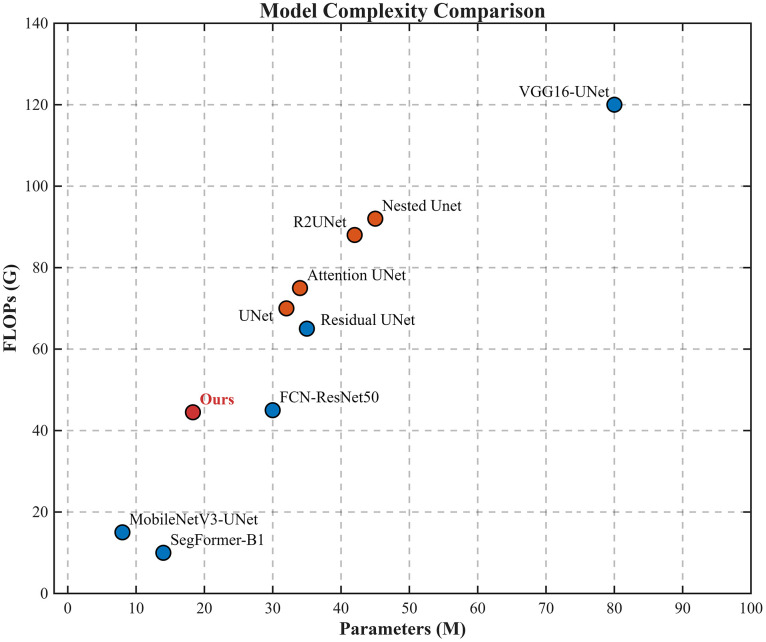
Model complexity comparison in terms of FLOPs and parameters.

Fig 10 compares the computational complexity of different segmentation networks in terms of FLOPs and parameter counts. Classic or non‑UNet models (blue circles) span a wide range of complexity: lightweight architectures such as MobileNetV3‑UNet and SegFormer‑B1 are located in the lower‑left region, while VGG16‑UNet lies in the upper‑right corner with the highest FLOPs and parameters. UNet variants (orange diamonds), including Residual UNet, Attention UNet, R2UNet and Nested UNet, are mainly distributed in the upper‑right area, showing that they generally incur larger computational and memory costs.

Our method (red square) is positioned noticeably to the left of most UNet variants, with only 44.5G FLOPs and 18.32M parameters. This complexity is much lower than that of typical UNet‑based models, yet, as shown in later experiments, the proposed network maintains competitive or superior segmentation performance. Therefore, the figure highlights that our model achieves a better balance between accuracy and efficiency, which is advantageous for deployment in resource‑constrained or real‑time applications.

#### 3.4.5 Statistical significance analysis.

To verify that the performance improvements of the proposed CrackNet are robust rather than resulting from random initialization, we conducted paired t-tests over repeated runs on all three datasets, with the corresponding p-values reported in Table 8. On the CFD dataset, both F1 and precision achieve strong statistical significance at α < 0.01 (p = 0.0043 and p = 0.0086, respectively), while recall is significant at α < 0.05 (p = 0.0187). These results indicate that the improvements on CFD are highly stable, though recall exhibits slightly higher variability due to the sensitivity of thin-crack detection to local noise. For the DeepCrack dataset, all three metrics show significance at α < 0.05, with precision and recall further reaching α < 0.01 (p = 0.0064 and p = 0.0079). This confirms that the proposed modules consistently reduce false positives and false negatives even under the challenging conditions and structural diversity in DeepCrack. On the Crack500 dataset, the improvements in F1 and recall are strongly significant at α < 0.01 (p = 0.0063 and p = 0.0011), while precision remains significant at α < 0.05 (p = 0.0152), reflecting expected fluctuations in false-positive rates across repeated runs. Overall, the statistical significance analysis demonstrates that the observed performance gains are consistent, reproducible, and not attributable to random chance, reinforcing the robustness and reliability of CrackNet across different datasets and crack characteristics.

**Table 8:** Statistical significance (p-values) of performance improvements based on paired t-tests over repeated runs. ✓ indicates significance at α = 0.05 or α = 0.01.

Dataset	Metrics	p-value	α < 0.05	α < 0.01
CFD	F1	0.0043	√	√
	Pr	0.0086	√	√
	Recall	0.0187	√	×
DeepCrack	F1	0.0236	√	×
	Pr	0.0064	√	√
	Recall	0.0079	√	√
Crack500	F1	0.0063	√	√
	Pr	0.0152	√	×
	Recall	0.0011	√	√

#### 3.4.6 Failure case analysis.

**Fig 11:**
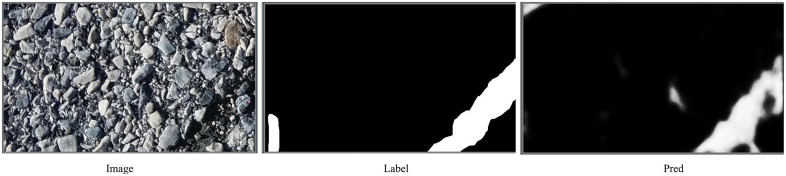
Representative failure case on complex textured pavement. From left to right: input image, ground-truth label, and predicted segmentation result.

Fig 11 presents representative failure cases of the proposed method, where the model produces incorrect predictions under challenging surface conditions. In this example, the original image contains highly textured aggregate patterns with strong intensity variations that visually resemble crack structures. Although the ground truth annotation indicates the presence of a crack only in a limited region, the model incorrectly predicts several false-positive responses in the surrounding textured background.

This failure can be attributed to the fact that coarse aggregates and stone boundaries exhibit elongated and high-contrast edges that are visually similar to cracks, making them difficult to distinguish using appearance cues alone. In such scenarios, the model tends to confuse crack-like textures with actual cracks, leading to over-segmentation. These cases highlight a current limitation of the proposed method when dealing with complex backgrounds containing strong texture noise. Incorporating additional contextual constraints or material-aware features may help alleviate this issue in future work.

#### 3.4.7 Exploring the impact of ECA attention on the LightMSCBlock module.

Table 9 presents the ablation study results comparing LightMSCBlock without and with the ECA module across three crack segmentation datasets. On the CFD dataset, the ECA version achieved a higher F1 (0.755) than the non-ECA version (0.7287), with only marginal differences in precision and recall, suggesting that ECA contributes to a more favorable balance between these metrics. For the DeepCrack dataset, LightMSCBlock with ECA achieved slightly higher precision (0.8654 vs. 0.8543) while maintaining similar recall, resulting in improved F1 (0.8214 vs. 0.8178). The effect was more pronounced on the Crack500 dataset, where both precision (0.8765 vs. 0.8664) and recall (0.7964 vs. 0.7776) increased, resulting in a higher F1 (0.7636 vs. 0.7553). These results indicate that incorporating ECA into LightMSCBlock generally improves segmentation performance across different datasets, with notable gains in more challenging scenarios, likely due to enhanced channel interaction and stronger feature representation.

**Fig 12:**
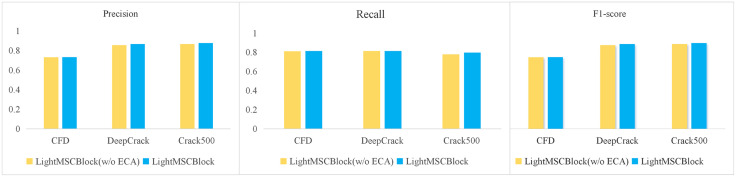
Performance Comparison of LightMSCBlock and LightMSCBlock(w/o ECA) across CFD, DeepCrack, and Crack500.

As shown in Table 9 and the visualization Fig 12, LightMSCBlock consistently outperforms LightMSCBlock(w/o ECA) across all three datasets in terms of Precision, Recall, and F1, demonstrating the effectiveness of the attention mechanism.

**Table 9:** Comparative experiments between different attention and SAF attention on three datasets.

	CFD			DeepCrack			Crack500		
Method	Pr	Recall	F1	Pr	Recall	F1	Pr	Recall	F1
LightMSCBlock(w/o ECA)	0.7304	0.8103	0.7287	0.8543	0.8125	0.8178	0.8664	0.7776	0.7553
LightMSCBlock	0.7325	0.8128	0.7550	0.8654	0.8126	0.8214	0.8765	0.7964	0.7636

## 4 Conclusions

With aging infrastructure and increasing concerns about structural safety, crack detection has become a critical component of structural health monitoring. Traditional manual inspection methods are time-consuming, subjective, and inefficient. At the same time, existing deep learning-based approaches, though promising, often struggle with accuracy and robustness under real-world conditions involving noise, lighting variations, and complex textures. Therefore, there is an urgent need for more accurate, robust, and practically deployable crack detection models to meet engineering demands.

To address these challenges, we propose CrackNet, a novel segmentation network tailored for concrete cracks, inspired by information theory to address uncertainty and maximize information flow. CrackNet integrates three modules: a Light multi-scale convolution enhancement block (LightMSCBlock) in the encoder for local and global feature capture, a SAF mechanism in skip connections for scale fusion and edge refinement, and a multi-scale feature fusion (MSFF) module in the decoder for enhanced integration. These designs improve feature clarity and reduce information loss. Experimental results on three public datasets—CFD, Crack500, and DeepCrack—demonstrate that CrackNet consistently outperforms baseline and state-of-the-art methods regarding accuracy, F1-score, and IoU, especially in noisy and cluttered scenarios.

Despite the notable improvements, the proposed model has several limitations. First, the relatively complex network structure increases computational cost, potentially hindering deployment on resource-constrained devices or in real-time applications. Second, the model may still struggle to detect fine cracks with low contrast or blurred edges, leading to potential false negatives. Third, this study primarily focuses on binary segmentation and does not include modules for extracting geometric crack features, such as width and length, which limits its application for quantitative damage assessment.

Future work will aim to (1) optimize the model’s architecture for lightweight and real-time performance through techniques such as efficient convolutions and knowledge distillation, (2) explore multi-modal fusion with additional data sources to improve detection robustness, and (3) integrate crack measurement and tracking components to extend the model from simple detection to comprehensive structural damage assessment. Additionally, we plan to explore potential extensions to other materials (e.g., asphalt or metal) or modalities (e.g., thermal imaging), which could provide new insights and further enhance the model’s generalizability and deployment in diverse real-world conditions. These improvements will contribute toward a more intelligent and complete structural health monitoring system.
